# Identification and Characterization of B-Cell Epitopes in the DBL4ε Domain of VAR2CSA

**DOI:** 10.1371/journal.pone.0043663

**Published:** 2012-09-06

**Authors:** Sisse B. Ditlev, Morten A. Nielsen, Mafalda Resende, Mette Ø. Agerbæk, Vera V. Pinto, Pernille H. Andersen, Pamela Magistrado, John Lusingu, Madeleine Dahlbäck, Thor G. Theander, Ali Salanti

**Affiliations:** 1 Centre for Medical Parasitology at the Department of International Health, Immunology and Microbiology, University of Copenhagen, and at Department of Infectious Diseases, Copenhagen University Hospital (Rigshospitalet), Copenhagen, Denmark; 2 National Institute for Medical Research, Tanga Medical Research Centre, Tanga, Tanzania; Universidade Federal de Minas Gerais, Brazil

## Abstract

Malaria during pregnancy in *Plasmodium falciparum* endemic regions is a major cause of mortality and severe morbidity. VAR2CSA is the parasite ligand responsible for sequestration of *Plasmodium falciparum* infected erythrocytes to the receptor chondroitin sulfate A (CSA) in the placenta and is the leading candidate for a placental malaria vaccine. Antibodies induced in rats against the recombinant DBL4ε domain of VAR2CSA inhibit the binding of a number of laboratory and field parasite isolates to CSA. In this study, we used a DBL4ε peptide-array to identify epitopes targeted by DBL4ε-specific antibodies that inhibit CSA-binding of infected erythrocytes. We identified three regions of overlapping peptides which were highly antigenic. One peptide region distinguished itself particularly by showing a clear difference in the binding profile of highly parasite blocking IgG compared to the IgG with low capacity to inhibit parasite adhesion to CSA. This region was further characterized and together these results suggest that even though antibodies against the synthetic peptides which cover this region did not recognize native protein, the results using the mutant domain suggest that this linear epitope might be involved in the induction of inhibitory antibodies induced by the recombinant DBL4ε domain.

## Introduction

*Plasmodium falciparum* induced malaria is a major cause of mortality and severe morbidity in large parts of the world, especially in sub-Saharan Africa. The majority of individuals, who die or become seriously ill from the disease, are young children and pregnant women. Previously immune women become susceptible to malaria during the first pregnancy [1]–[3]. The disease is caused by sequestration of *P. falciparum*-infected erythrocytes (IE) in the placenta, mediated through the parasite ligand VAR2CSA, which binds to the placental receptor chondroitin sulfate A (CSA). Placental malaria (PM) is a major cause of maternal anemia, fetal growth retardation, stillbirth and delivery of low-birth-weight babies [3]. Women in high malaria transmission areas acquire antibodies against CSA-binding parasites as a function of parity, which explains why first-time pregnant women are most susceptible to malaria [4]. VAR2CSA is a member of the *Plasmodium falciparum* erythrocyte membrane protein 1 (PfEMP1) family, which is encoded by the *var* genes [5]–[7]. Even though interclonal variation in the *var2csa* gene is low compared to other *var* genes, variability is still found within *var2csa* which presents a challenge for vaccine development [8]. IgG acquired during pregnancy, recognize CSA-binding parasites of diverse geographical origin [9], [10]. This suggests that conserved VAR2CSA protective epitopes exist and identification of such epitopes could be useful in PM vaccine development. The full-length ecto-domain of VAR2CSA is a large antigen (350 kDa) and thus difficult to use as a recombinant vaccine. It is therefore needed to define smaller region(s) of the VAR2CSA that can induce antibodies capable of inhibiting parasite binding to CSA. We have previously shown that antibodies raised against a recombinant protein including the Duffy-Binding-Like-4ε (DBL4ε) of VAR2CSA from the FCR3 strain effectively inhibit homologous IE binding to CSA. We have further demonstrated cross-inhibition of heterologous parasites using antibodies against the DBL4ε domain [11]. Whether the cross-reactivity of antibodies against recombinant DBL4ε is caused by conserved epitopes or by overlapping polymorphism between heterologous parasite isolates, is currently not known. In this study, we have applied a peptide array covering the DBL4ε domain with the aim of identifying regions that are targets of the induced inhibitory antibodies. By narrowing down the regions that are responsible for the induction of the inhibitory antibodies it may be feasible to define sero-variants of VAR2CSA that could be included in a multivalent vaccine. In addition, it would be possible to remove immuno-dominant B-cell epitopes that are not part of the protective response, in order to focus the immune response towards the significant epitopes.

Our objectives in this study were: (i) to identify DBL4ε epitopes that are targeted by DBL4ε-specific antibodies, which inhibit CSA-binding of parasites, and to define DBL4ε peptides which are able to induce antibodies that (ii) recognize the native protein and (iii) prevent parasite binding to the placental receptor CSA.

## Results

### Prediction of linear B-cell epitopes in the DBL4ε-FCR3 domain

Parameters such as hydrophobicity, chain flexibility and polarity of polypeptide chains can be correlated to the location of linear B-cell epitopes. We used BepiPred to predict B-cell epitopes in the DBL4ε-FCR3 domain. (http://www.cbs.dtu.dk/services/BepiPred/) [12]

Five B-cell epitopes were identified:

Epitope 1: YNPTGKGIDDANK, Epitope 2: GSSNTNDIDTKRARTDWWENETITNGTDRK, Epitope 3: KSKCDPPKRADTCGDNSNI, Epitope 4: RKSNKESEDGKD and Epitope 5: AYNTTSGTVNKKLQKKETECEEEKGPLD. The predicted B-cell epitopes 1–5 are indicated on the peptide array used in this study (Figure S1A). Epitopes 1–4 locate to loop regions on a structural model of DBL4ε-FCR3 (Figure S1B). Epitope 5 is located in a region flanking the sequence that was used to make the structural model and is therefore not mapped on the model.

### Identification of epitopes targeted by inhibitory DBL4ε-specific sera

**Figure 1:**
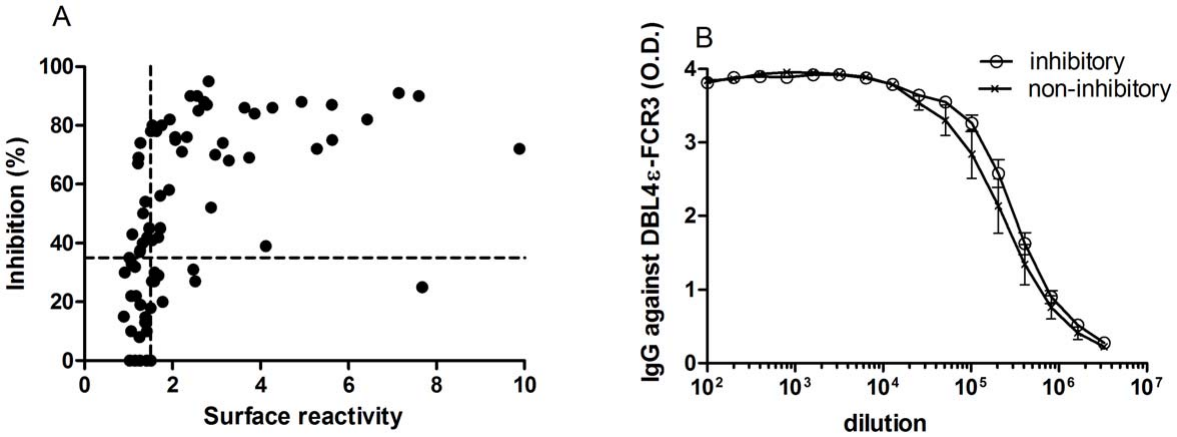
Correlation between the ability of different DBL4ε anti-sera to react with native VAR2CSA expressed on infected erythrocytes (IE) and their ability to inhibit the binding of VAR2CSA-expressing FCR3 IE to CSA. (A) Anti-DBL4ε sera (n = 75) from rats were tested for reactivity in FACS and for ability to inhibit parasite binding to CSA in a static binding assay. (B) Comparison of anti-VAR2CSA antibody levels in sera which inhibited binding (N = 15) and sera without anti-binding activity (N = 9). Antibody mean titre was determined as EC50 using GraphPad Prism5.

**Figure 2:**
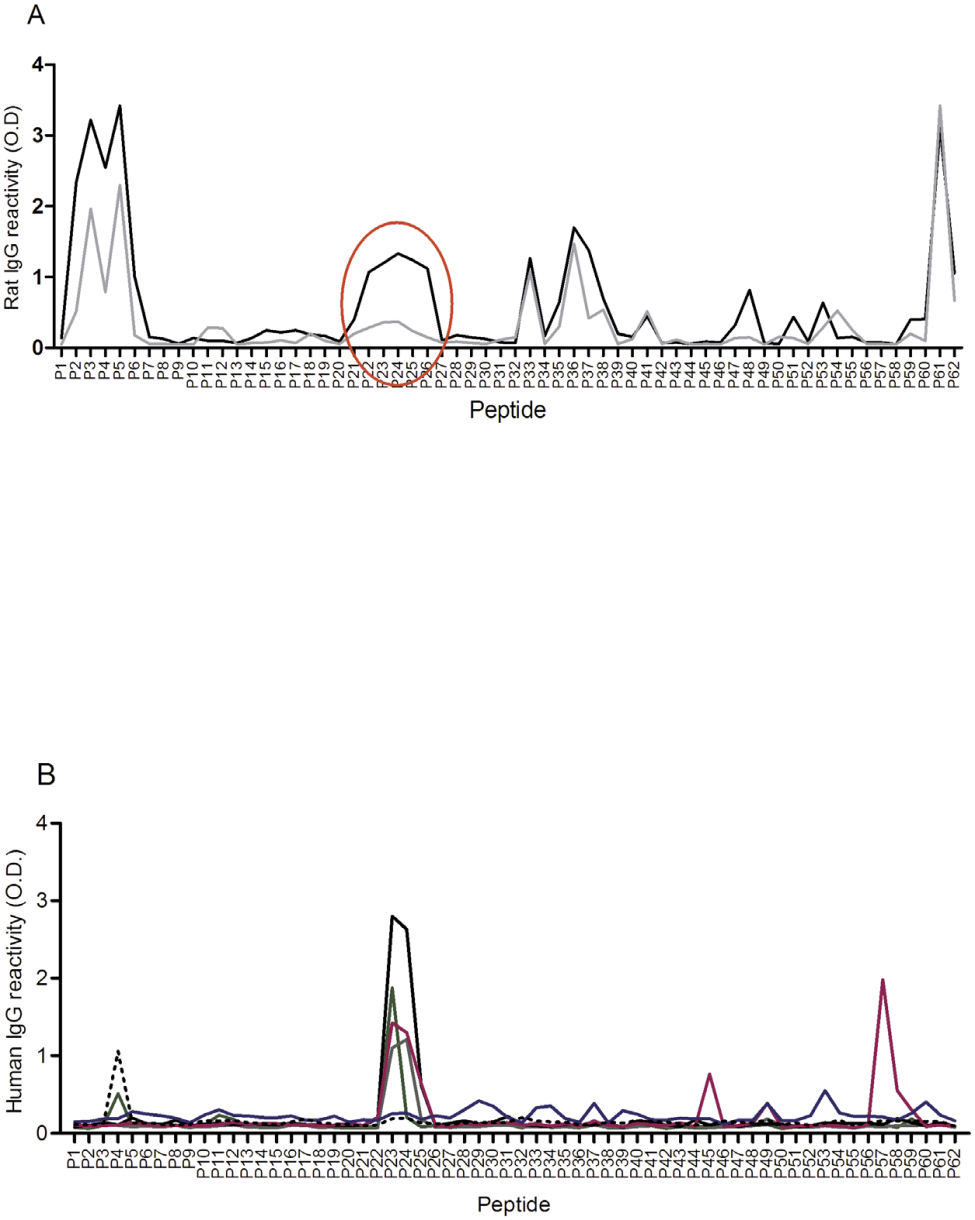
Reactivity of anti-sera to 62 overlapping DBL4ε peptides measured by ELISA. (A) Reactivity of DBL4ε anti**-**sera to each peptide is shown as the average O.D. values for the inhibitory anti-sera (black line n = 47), and the non-inhibitory anti-sera (grey line n = 28). The red circle highlights the largest region of consecutive peptides with the highest difference in peptide binding of the binding inhibitory anti sera and the non-inhibitory anti-sera. (B) O.D. values for reactivity of human sera from hyper-immune women (*n* = 6) to each of the 62 overlapping peptides covering the DBL4ε-FCR3 domain. Each colored line represents one female sera sample.

**Figure 3:**
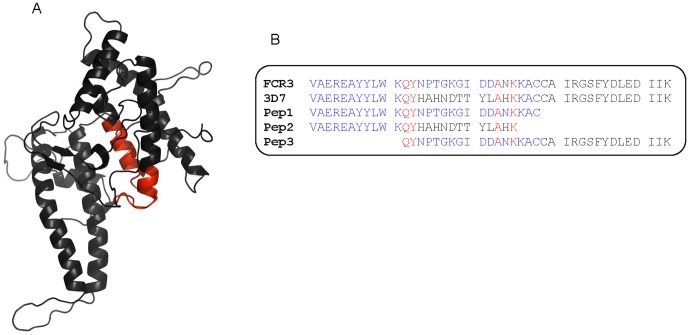
Mapping peptide-region on the DBL4ε model and sequence alignment. (A) Peptide-region P22–P26 mapped on a DBL4ε-FCR3 model. (B) FCR3 and 3D7 sequences aligned with 3 peptides produced for immunization.

We have previously shown that the induction of DBL4ε-specific antibodies that inhibit binding of IE to CSA is dependent on the number of immunizations, the VAR2CSA genotype, the species of animals and the size of the recombinant protein [11], [13]–[16]. From these studies, we had 82 DBL4ε-specific anti-sera available from animals immunized with recombinant DBL4ε, DBL4ε-ID4 or double domains containing DBL4ε protein with either FCR3, 3D7, HB3 or 7G8 genetic background. This panel of DBL4ε-specific sera was tested for the ability to recognize native VAR2CSA on the surface of FCR3 parasites and the ability to inhibit parasite binding to CSA (Figure 1A). We found a positive correlation between parasite surface reactivity and inhibitory capacity of the sera using Spearman correlation test (r = 0.66, P<0.0001). EC50 analysis was performed on 24 samples (15 samples with highest inhibitory capacity, ≥80% inhibition and 9 non-inhibitory samples, ≤10% inhibition) against the DBL4ε-FCR3 protein to examine whether the difference in anti-adhesive capacity was exclusively due to differences in IgG titers (Figure 1B). Figure 1B shows the level of IgG by using EC50 analysis of (the inhibitory and non-inhibitory group) and there is no apparent difference. To define potential epitopes involved in the inhibitory response we synthesized 63 overlapping peptides covering the entire DBL4ε. The 82 DBL4ε-anti-sera identified were arbitrarily divided into those which were highly inhibitory (35 samples, >67% inhibition) and those which were weakly inhibitory (47 samples, ≤42% inhibition) and analyzed for reactivity with the DBL4ε peptides. Figure 2A shows the average reactivity of the inhibitory sera (black line) and the non-inhibitory sera (grey line) measured as optical density in a peptide-ELISA. The most immunogenic linear regions of the DBL4ε- domain were covered by peptides P2–P6, P21–P26, P33, P35–P38, P41, P47–48, P51, P53–P55 and P59–P62. Interestingly, these regions, except P2–6, P33 and P41 were also identified as linear B-cell epitopes *in silico* (Figure S1). The ELISA-identified epitope P21–P26 is located in the BepiPred predicted Epitope 1, the P33–P38 epitope in BepiPred Epitope 2, the P47–P48 epitope in BepiPred Epitope 3, the P51–P54 epitope in BepiPred Epitope 4 and the P59–P62 epitope in BepiPred Epitope 5. The peptides were grouped into 11 regions (P1, P2–P7, P8–P21, P22–P26, P27–P32, P33–39, P40–P45, P46–P49, P50–P58 and P59–P63 based on the profile in Figure 2A. The correlation between the reactivity of anti-DBL4ε sera to the peptide-regions and the ability of the sera to inhibit parasite binding were investigated by univariate and multivariate regression, with differences in parasite strain and borders taken into account in the model (Table 1). Reactivity against P2–P7, P22–P26 and P47–P49 were significantly associated with the inhibitory capacity of the sera (Table 1). To compare the DBL4ε-induced antibody response with naturally acquired antibodies against VAR2CSA, sera from six multi-gravid Tanzanian women in their 3rd to 11th pregnancy, were analyzed for reactivity to the DBL4ε peptides. The six serum samples were selected from a panel of Tanzanian sera based on reactivity to VAR2CSA expressing parasites and reactivity to the recombinant full-length VAR2CSA protein (data not shown). Four of the immune sera showed high reactivity to the peptide-region P23–P25: YNPTGKGIDDANKKACCAIRGS (Figure 2B) and the same samples inhibited the CSA-binding of VAR2CSA expressing FCR3-parasites by 40%–80% (data not shown). From Figure 2 it is evident that antibodies are induced against peptide-region P22–P26 in rats and the central part of this region (P23–P25) is also a target in natural infections of pregnant women. This difference could be due to human MHC restriction or more likely different presentation of the loop in natural VAR2CSA compared to recombinant DBL4 subunit vaccine. Based on these findings we chose to further characterize the region comprised of the peptide region P22–P26. The peptide region P22–P26 corresponds to residues 88–115 in the recombinant DBL4ε FCR3 protein (Table S1). By mapping these peptides on a DBL4ε-FCR3 structure model it is apparent that this region is situated in a loop with flanking α-helices in the S2-subdomain (Figure 3A). A multiple alignment was done on 30 *P. falciparum* sequences, some derived from GenBank and some sequenced using primers targeting conserved regions on either side of the P22–P26 peptide-region. We found the B-cell epitope covered by peptides P22–P26 to fall into two highly conserved cassette types (Figure 3B and Figure S2). 22 of the 30 sequences fall in to the FCR3-type and 8 in the 3D7-type (Figure S2) [17].

**Table 1:** Univariate and multivariate stepwise backwards linear regression analyses.

		Univariate analysis			Multivariate analysis	
Peak	Coeff.	95% CI	p-value	Coeff.	95% CI	p-value
P1	0.035	[−0.260–0.329]	0.816	NS	NS	NS
P2–P6	0.024	[0.012–0.035]	0.000	0.016	[0.004–0.029]	0.010
P7–P20	0.038	[−0.015–0.091]	0.160	NS	NS	NS
P21–P26	0.018	[0.005–0.031]	0.007	0.013	[0.0005–0.026]	0.042
P27–P32	0.007	[−0.112–0.126]	0.907	NS	NS	NS
P33–P38	0.023	[0.002–0.044]	0.032	NS	NS	NS
P39–P46	0.021	[−0.043–0.085]	0.519	NS	NS	NS
P47–P49	0.094	[0.023–0.165]	0.010	0.076	[0.009–0.143]	0.028
P50–P58	0.024	[−0.014–0.061]	0.207	NS	NS	NS
P59–P62	0.018	[−0.012–0.047]	0.238	NS	NS	NS

The table shows the association between the ability of DBL4ε anti-sera to inhibit binding of VAR2CSA expressing infected erythrocytes to CSA and the level of antibodies to the individual peptides. Antibody reactivity to groups of DBL4ε peptides according to the reactivity shown in figure 2A. NS: Not Significant.

### Potency of peptide-specific antibodies raised in rats

**Figure 4:**
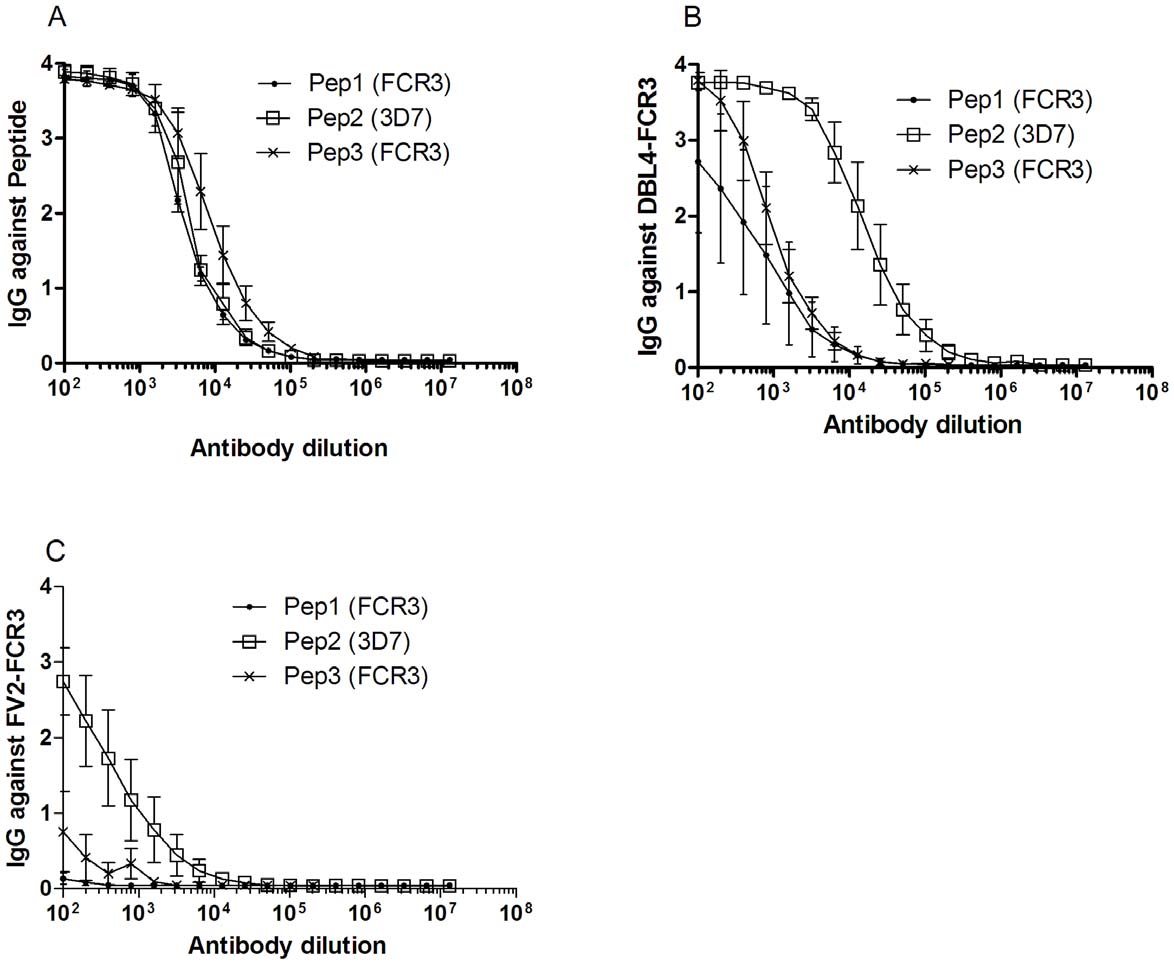
Reactivity of peptide-specific antibodies with the homologous peptide, recombinant DBL4ε and full-length VAR2CSA (FV2) from FCR3. (A) Serial two-fold dilution of rat antibodies specific for Pep1, Pep2 and Pep3 was tested in ELISA against the homologous peptide (B) and the recombinant proteins: DBL4ε-FCR3 and (C) FV2-FCR3.

Based on the FCR3 sequence we designed two overlapping peptides covering the regions P19–P23 (Pep1; VAEREAYYLWKQYNPTGKGIDDANKKAC) and P22–P28 (Pep3; QYNPTGKGIDDANKKACCAIRGSFYDLEDIIK). A peptide covering P19–P23 was also designed based on the 3D7 sequence (Pep2; VAEREAYYLWKQYHAHNDTTYLAHK) (Figure 3B). Three rats were immunized with each of the KLH-conjugated peptides and the immune sera were analyzed for reactivity in ELISA against corresponding peptides, the DBL4ε domain and against the full-length VAR2CSA protein (FV2). All three peptide immunizations resulted in high antibody titers against corresponding peptide (Figure 4A) and to varying degree against the recombinant DBL4ε protein and full-length VAR2CSA (Figure 4B and 4C). Of the three peptides, Pep2, based on the 3D7 sequence showed highest reactivity against full-length FCR3 VAR2CSA (Figure 4C), and against DBL4ε (Figure 4B). The peptides based on the FCR3 sequence, Pep1 and Pep3, induced antibodies that reacted with the recombinant DBL4ε protein (Figure 4B) but not with the full-length VAR2CSA protein (Figure 4C).

### Reactivity against native VAR2CSA protein and inhibition of parasites to CSA by peptide-specific antibodies

**Figure 5:**
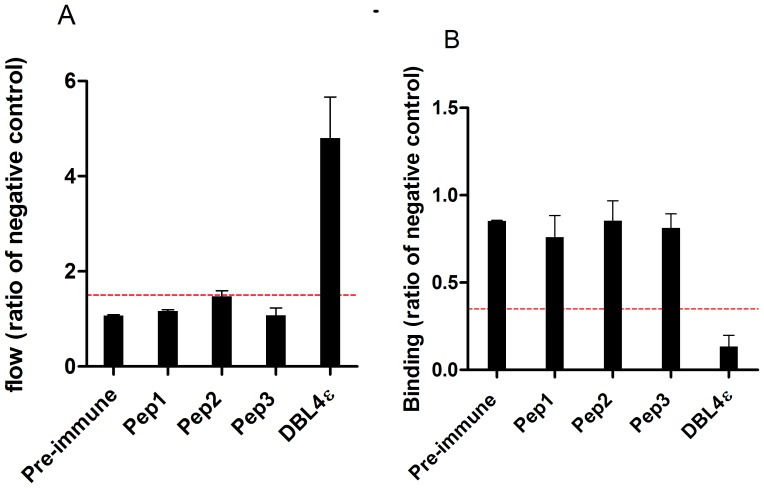
Reactivity of peptide-specific antibodies against native VAR2CSA and their ability to inhibit binding of VAR2CSA expressing infected erythrocytes to CSA. (A) Reactivity against native VAR2CSA expressed on the surface of FCR3-IE was measured by flow cytometry and is presented as the ratio of the negative control. A ratio >1.5 is considered positive, indicated with the red broken line. (B) Inhibition of parasite binding to CSA using sera (1∶10 dilution) from rats immunized with a KLH-conjugated peptide or DBL4ε-FCR3. Inhibition/binding is presented as the ratio of binding compared to the negative control (sera specific for a non-VAR2CSA protein, DBL3γVAR1) ((Binding [test sample])/(Binding [negative control])). Binding values below 0.35 is considered as inhibitory and indicated by the red broken line.

The peptide-specific sera were analyzed for the ability to bind native VAR2CSA on the surface of parasite-infected erythrocytes (FCR3-IE) and for inhibition of CSA-binding of FCR3-IE. Of the three peptide-specific sera, only the Pep2 antibodies showed some surface reactivity, although at very low levels compared to the DBL4ε-induced antibodies (Figure 5A). The ability of the peptide-specific sera to inhibit binding of FCR3-IE to CSA was measured in a static binding assay. None of the sera were able to effectively block parasite binding to CSA (Figure 5B).

### Comparison of surface reactivity and inhibition of FCR3-IE adhesion to CSA by antibodies specific for wild-type and for mutated DBL4ε protein

**Figure 6:**
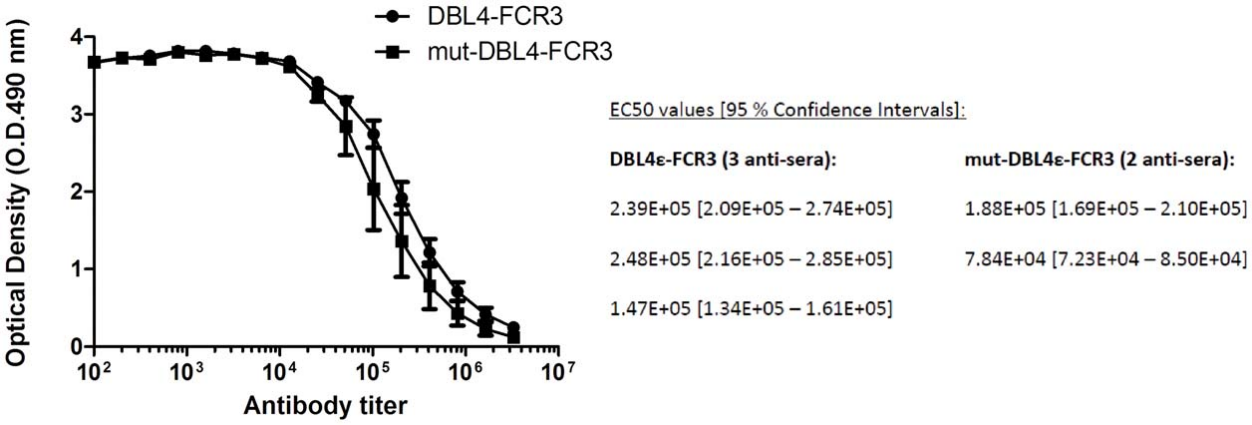
Antibody response of sera specific for wild-type DBL4ε-ID4 and the mut-DBL4ε. Serial two-fold dilution of rat anti-DBL4 and anti-mut-DBL4 polyclonal sera (initial dilution: 1/100) tested in ELISA against recombinant DBL4-FCR3. Antibody mean titer was determined as EC50 using GraphPad Prism5.

**Figure 7:**
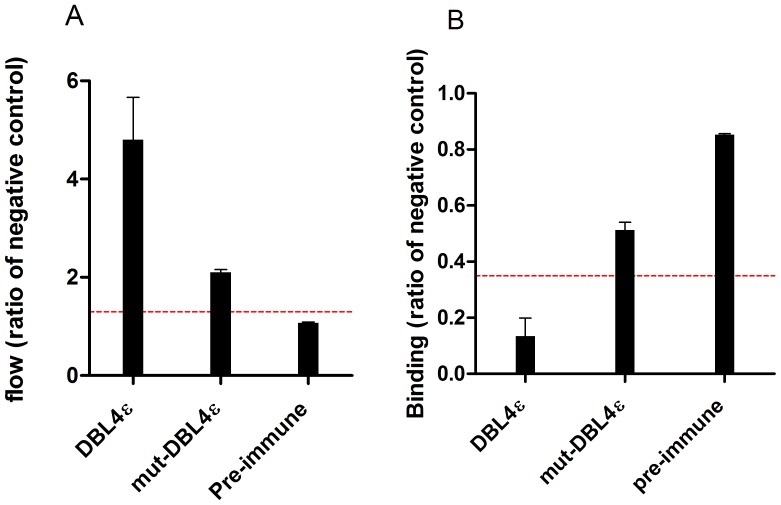
Antibody reactivity against native VAR2CSA expressed on the surface of FCR3-IE and inhibition of FCR3-IE adhesion to CSA. (A) Anti-sera from rat immunized with either wild-type DBL4ε-FCR3 or mut-DBL4ε-FCR3 were tested for reactivity against native VAR2CSA expressed on the surface of FCR3-IE using FACS (IFA) (ratio to negative control). Pre-immune sample was used as negative control. A ratio >1.5 is considered positive, indicated with the red broken line. (B) Anti-sera (1∶10 dilution) from rat immunized with either DBL4ε or mut-DBL4ε were tested using FCR3-IE for the ability to inhibit the binding to CSA in a 96-well plate assay. The data is the ratio of binding compared to negative control (DBL3γVAR1). ((Binding [test sample])/(Binding [negative control])). Binding values below 0.35 is considered as inhibitory and indicated by the red broken line.

Immunization of rats using the designed peptides (Pep1, Pep2, Pep3) covering P22–P28 and P19–P23 in the DBL4ε peptide array did not result in antibodies capable of inhibiting parasite binding to CSA. This could be due to wrong folding of the long synthetic peptides. In order to unravel the role of this loop region in the induction of inhibitory antibodies induced by DBL4ε immunization, we designed a recombinant DBL4ε protein lacking the specific loop region (mut-DBL4ε). With the DBL6ε as template, a structural alignment of DBL4ε and DBL6ε was used to identify seven amino acids in DBL4ε that could be removed without destroying the integrity of the intrinsic DBL structure. Accordingly we removed seven amino acids in the DBL4ε-S2 loop region: PTGKGID, expressed the DBL4ε mutant protein in insect cells and immunized rats. DBL4ε-FCR3 and mut-DBL4ε-FCR3 induced comparable antibody titers; mut-DBL4ε: 7.84E+04-1.88E+05 and DBL4ε: 1.47E+05-2.48E+05 (Figure 6). The antibodies induced by the mut-DBL4ε protein were clearly reactive against the native VAR2CSA protein on IE, but showed only half the reactivity as the DBL4ε induced antibodies (Figure 7A). In addition, the ability to induce inhibitory antibodies was reduced when using the mutated DBL4ε protein compared to the wild-type protein (Figure 7B).

## Discussion

Naturally acquired immunity against placental malaria is acquired in highly endemic areas. Women acquire protective antibodies as a function of parity thus it is mainly in the first pregnancy women present severe symptoms. One of the major challenges in the development of a PM vaccine is the sequence polymorphisms within the VAR2CSA antigen. We have previously shown that antibodies induced against the VAR2CSA DBL4ε-FCR3 protein can inhibit a broad panel of genotypically different fresh placental isolates [11]. The DBL4ε is not part of the CSA-binding domain as this region has been identified in the N-terminal part of the VAR2CSA as ID1-ID2a [18], [19]. The mode of action and the specific target of the binding-inhibitory DBL4ε antibodies induced in rats are currently not known. In this study, we utilized the panel of sera with high binding-inhibitory and low binding-inhibitory capacities to map epitopes potentially involved in the inhibitory response. By analyzing this panel of sera on an array consisting of peptides spanning the entire DBL4ε-FCR3 domain we identified a linear region showing differential recognition between sera with inhibitory and non-inhibitory capacity. Prominently, we show that the central part of this peptide region is also targeted by naturally acquired IgG. Mapping the peptide on a structure model of DBL4ε-FCR3 suggests the region to be located around a loop structure in sub-domain S2. [18]. This loop region is found to be highly conserved but appears as two different variants (Figures 3B and S2). Peptides designed to cover this loop structure were all immunogenic and elicited a potent antibody response against the immunized peptide itself and against the recombinant DBL4ε-FCR3. However, this did not apply to the recombinant FV2-FCR3 or native VAR2CSA expressed on IE. These linear peptides are most likely a part of a larger conformational epitope, which would explain why the whole DBL4ε domain is needed to induce a proper inhibitory antibody response. Alternatively, the native epitopes in this loop region could not be reconstructed as synthetic peptides. The fact that we found the antibodies against the 3D7 peptide to recognize the heterologous recombinant protein (of FCR3-type) better than the homologous FCR3 peptide antibodies, could be due to a difference in the quality and folding of the peptides.

IgG induced against a mutated DBL4ε-FCR3 protein deleted for the identified loop region were less reactive against native VAR2CSA on IE compared to IgG induced against wild-type DBL4ε-FCR3. The ability of the mutated DBL4ε-FCR3 to induce inhibitory IgG was also reduced compared to wild-type DBL4ε. The relatively large reduction in surface reactivity and inhibitory capacity suggest that the removed B-cell epitope plays an important role in the induction of inhibitory antibodies. However, it cannot be ruled out that the deletion of the epitope has affected the overall fold of the DBL protein and thereby affected the folding of distant B-cell epitopes. We have previously shown that IgG induced against DBL4ε-FCR3 can inhibit around 75% of field isolates adhesion to CSA [11]. The fact that the epitopes targeted by inhibitory DBL4ε IgG are recognized in 75% of sequenced isolates could imply that sequence variation in this area plays a central role for the efficacy of a DBL4ε-FCR3 vaccine. Whether it is conserved epitopes or overlapping polymorphism between heterologous parasites that are recognized is not known. As the DBL4ε is a very potent inducer of binding-inhibitory antibodies, although it is not part of the CSA-binding region [18], [19,19], it will be attractive to combine B-cell epitopes from DBL4ε with VAR2CSA CSA binding regions in a single vaccine. This approach will potentially induce antibodies that act synergistically, and provide a more effective inhibition of parasite binding to CSA by 1) targeting the direct binding region by ID1-ID2a antibodies and 2) by steric hindrance mediated by DBL4ε antibodies.

## Materials and Methods

### Ethics statement

All procedures regarding animal immunizations complied with European and National regulations. This study was approved by the Danish animal welfare council under the Danish Ministry of Justice Approval ID: 2008/561-1498. The Tanzania Medical Research Coordinating Committee approved ethical clearance to conduct malaria related studies in pregnant women of northeastern Tanzania (NIMR/HQ/R.8a/Vol. IX/559). Written informed consent was obtained from each of the participating pregnant women from Korogwe Tanzania.

### Protein production

The DBL4 antigens were based on native FCR3, 3D7, HB3 and 7G8 *var2csa* and cloned from genomic *P. falciparum* DNA and produced with either amino acid number: 1583-1947 (364 aa) or: 1583-1989 (407 aa). The gene fragments were cloned into the *Baculovirus* vector pAcGP67-A (BD Biosciences) with a C-terminal V5 tag and a histidine tag. Linearized Bakpak6 baculovirus DNA (BD Biosciences) was co-transfected with pAcGP67-A into Sf9 insect cells. The histidine-tagged recombinant protein was purified on Ni2+ sepharose columns from supernatant of baculovirus infected High-Five insect cells in ÄKTA-express purification system (GE-Health-care). Plasmid sequencing was done by Macrogen, Korea.

### Peptide synthesis and KLH conjugation

The peptide array consisted of 63 synthetic 16–28 amino acids long overlapping peptides, spanning amino acids 25–407 of the FCR3 DBL4 domain. The peptides were prepared by Schafer-N Copenhagen and the purity of the peptides was expected to be 70% or higher. The carrier peptides were activated using 3-maleimidopropionic acid *N*-hydroxy-succinimide ester (MPS) as the cross-linking. Maleimide-KLH contains approximately 80 maleimide groups per KLH molecule.

### Animal immunizations

Rat anti-sera were produced in Wistar rats (outbred) (Taconic, Denmark) by subcutaneous injection of 20 µg/ml KLH-conjugated peptide or 30 µg/ml recombinant protein in Freund's complete adjuvant, followed by two boost injections of 10 µg/ml KLH-conjugated peptide or 15 µg/ml recombinant protein in Freund's incomplete adjuvant at three-weeks intervals. Immune-serum was collected eight days after the final boost injection and was stored at −20°C until use. All immunizations induced antibodies against the KLH-conjugated peptide as measured by enzyme-linked immune-sorbent assay (ELISA) of the final bleed.

### Peptide ELISA

ELISA plates (Nunc) were coated overnight with dissolved peptide (3 µg/ml) or recombinant protein (1 µg/ml) in PBS at 4°C. Plates were incubated with blocking buffer for 1 hour at room temperature (RT) to inhibit non-specific binding to the plate. The rat sera samples were diluted in blocking buffer (1∶100), added to the wells, and incubated for 1 hour at RT. Plates were washed three times in PBS between the different steps. Visualization of the binding of the rat sera IgG was done by adding HRP-conjugated anti-rat IgG (A9037 Sigma-Aldrich). The plates were incubated for 1 hour with the anti-rat IgG antibody diluted 1∶3000 in blocking buffer. The colour reactions were developed for 15 min by adding o-phenylenediamine substrate and stopped by adding 2.5 M H_2_SO_4_. The optical density was measured at 490 nm in an ELISA plate reader (VersaMax, Molecular Devices). Antibody titers were determined from serial two-fold dilutions of individual sera and represent the reciprocal dilution at the 50% effective concentration (EC50) established using non-linear regression to fit a variable slope sigmoidal equation to the serial dilution data. For determination of ELISA EC50 a four-parameter logistic regression was used to fit the variable slope sigmoidal equations to the serial dilution data using GraphPad Prism5.0 (GraphPad Software, Inc., San Diego, CA).

### Statistical analysis

The association between the ability of an anti-serum to inhibit parasite binding and the ability to react with the native protein on the surface of infected erythrocytes was investigated by Spearman's rank correlation test. The association between an anti-serum ability to react with peptides and the ability to inhibit parasite binding was analysed using univariate and multivariate linear regression. Based on the profile of antibody responses to the peptides in the peptide array, antibody reactivity was assigned to 10 regions based on the average reactivity of the anti-serum to the peptides constituting the region. The association was first tested in univariate models using inhibition of parasite binding as dependent variable and region reactivity as independent variable. Regions for which the p value for this association was <0.2 was then entered into a multivariate analyses and backward stepwise elimination strategy was employed. According to this strategy, the regions, which showed the least association with parasite binding inhibition were removed one by one, until we reached a multi-linear model incorporating the results for the regions to which antibody levels were statistically significantly associated with binding inhibition. Parasite strains and construct border were corrected for in all models. P<0.05 was considered significant. Stata 12 software was used for the statistical analyses.

### Modeling the structure of the DBL4ε -FCR3 of the VAR2CSA domain

The modeling of the DBL4ε-FCR3 was done as described previously on the 3D7 variant [20]. In brief, the structure of the DBL4ε-FCR3 domain was modeled using the HHpred server with default settings [21]. The HHpred method is based on comparisons and alignments of hidden Markov models (HMMs), which include gaps and insertion probabilities. Template alignments proposed by the HHpred method were used to generate 3D models by using a HHpred server toolkit protocol for MODELLER [22]. The structural visualization was produced using PyMol (Available: http://www.pymol.org/).

### *P. falciparum* isolate and culture

The laboratory parasite strain FCR3 of *P. falciparum* was used. Parasite culture was performed as described elsewhere [23]. In brief, the parasites were grown in culture using 5% hematocrit of human blood group 0 Rh+ in RPMI 1640 medium supplemented with 25 mmol/l sodium bicarbonate (Sigma-Aldrich), 0.125 µg/ml Gentamycin, 0.125 µg/ml Albumax II (Invitrogen) and 2% human sera (NHS). The parasite culture was regularly panned on human choriocarcinoma cell line BeWo to select for CSA binding [23]. The culture was free of Mycoplasma spp., which was determined by PCR.

### Flow-cytometry

The ability of anti-peptide antibodies to recognize the VAR2CSA antigen on the parasites was measure by flow-cytometry (FCM) using a protocol modified from [23]. Briefly, the parasite culture was enriched for late trophozoite and schizont stage parasites in a strong magnetic field (MACS, Miltenyi). Aliquots of 2×10^5^ IE in a total volume of 100 µl were labeled by ethidium bromide and sequentially exposed to (1) 15 µl animal serum and (2) 1∶100 dilutions of FITC labeled secondary antibodies specific for IgG from the individual species: Mouse (FL2000, Vector); rat (62–9511, Invitrogen) and rabbit (FL1000, Vector). As a negative control, IE were incubated both with sera from non-immunized animals and without animal serum and then exposed to secondary antibodies specific against IgG from different animal species. Data from 5000 infected cells (ethidium bromide positive) was collected on a FC500 Flow Cytometer (Beckman Coulter). The median FITC fluorescence intensity was determined using Winlist Software (Verity Software House).

### Assay for inhibition of parasite binding

The ability of the immunization induced antibodies to prevent parasite binding to CSA was measured using a parasite-binding-assay. 2×10^5^ tritium-labelled late-stage IE and 15 µl sera in a total volume of 120 µl were added in triplicates to wells coated with 2 µg/ml of the commercially available chondroitin sulfate proteoglycan Decorin (D8428; Sigma-Aldrich). After 90 min incubation at 37°C, unbound erythrocytes were washed away by resuspension by a pipetting robot (Beckman Coulter). Adhering of IE was determined by liquid scintillation counting on a Topcount NXT (Perkin-Elmer).

### Construction of DBL4ε mutant

The mutant DBL4ε construct was designed with a smaller loop region in S2 sub-domain. The construct was produced by cloning of each fragment on either side of the loop-depletion with a flanking region on each side of the deletion and joined together as a new construct; mut-DBL4ε-FCR3. With DBL6ε-FCR3 as template, alignment of the sequences for the two constructs shows seven amino acids more (**PTGKGID**) between amino acid 112 and 120 in the DBL4ε- construct than in the DBL6ε construct. The primers used for producing the mutant DBL4ε covered 15 bases on each side of the 7 deleted amino acids. The primers used were: Forward: TGGAAACAGTATAATGATGCGAATAAGAAA and Reverse: TTTCTTATTCGCATCATTATACTGTTTCCA. Aligning the DNA sequences from the mutated DBL4ε construct with the original DBL4ε construct confirmed the deletion of 21 nucleotides corresponding to the seven amino acids PTGKGID.

## Supporting Information

Figure S1 **Prediction of B-cell epitopes and mapped on a DBL4ε-FCR3 model.** (A) The five B-cell epitope predicted by BepiPred and mapped on the 63 overlapping DBL4ε-FCR3 single peptides in different colors. (B) Four of the five predicted B-cell epitopes mapped on the DBL4ε-FCR3 model. Epitope 5 is located in a sequence outside the boarders used for modeling the DBL4ε domain. Red = epitope 1, blue = epitope 2, green = epitope 3, yellow = epitope 4 and purple = 5.
(TIF)

Figure S2 **Comparison of 30** ***P. falciparum*** **sequences covering the polymorphic region of peptide-region P22–P26 by alignment using BioEdit.**
(TIF)

Table S1 **Amino acid sequence of each peptide and corresponding amino acid position in the DBL4ε-FCR3 domain.**
(DOCX)
