# Supplementary figures and images for: Identification and Characterization of B-Cell Epitopes in the DBL4ε Domain of VAR2CSA
Source: PLoS One. 2012 Sep 6;7(9):e43663. doi: 10.1371/journal.pone.0043663 (PMC3435390; doi:10.1371/journal.pone.0043663)

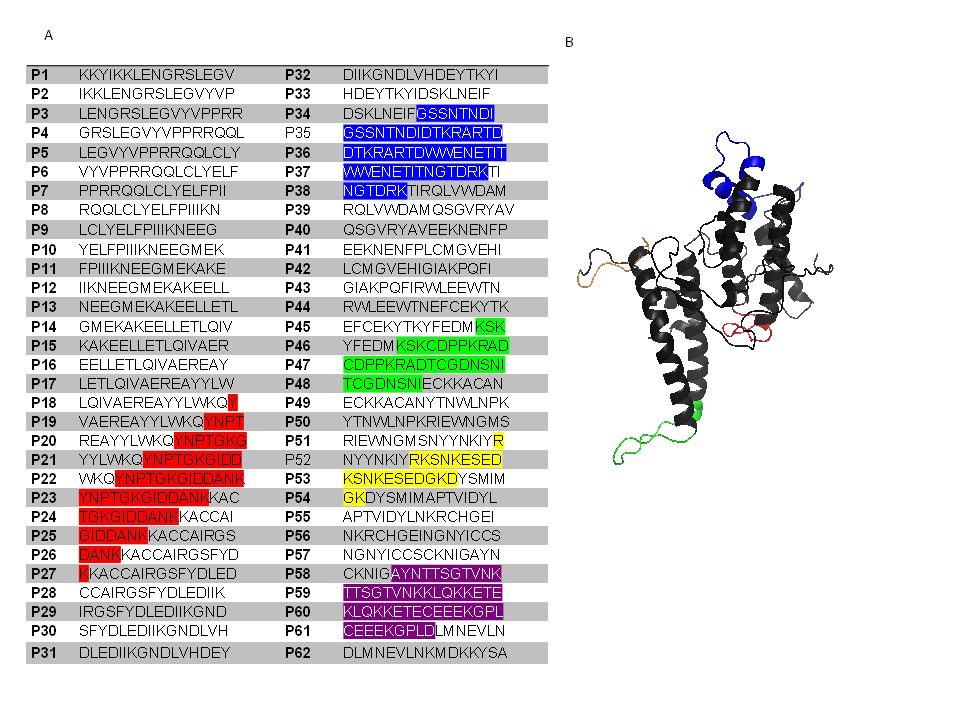

Supplement: Figure S1 — Prediction of B-cell epitopes and mapped on a DBL4ε-FCR3 model. (A) The five B-cell epitope predicted by BepiPred and mapped on the 63 overlapping DBL4ε-FCR3 single peptides in different colors. (B) Four of the five predicted B-cell epitopes mapped on the DBL4ε-FCR3 model. Epitope 5 is located in a sequence outside the boarders used for modeling the DBL4ε domain. Red = epitope 1, blue = epitope 2, green = epitope 3, yellow = epitope 4 and purple = 5. (TIF) [file pone.0043663.s001.tif]

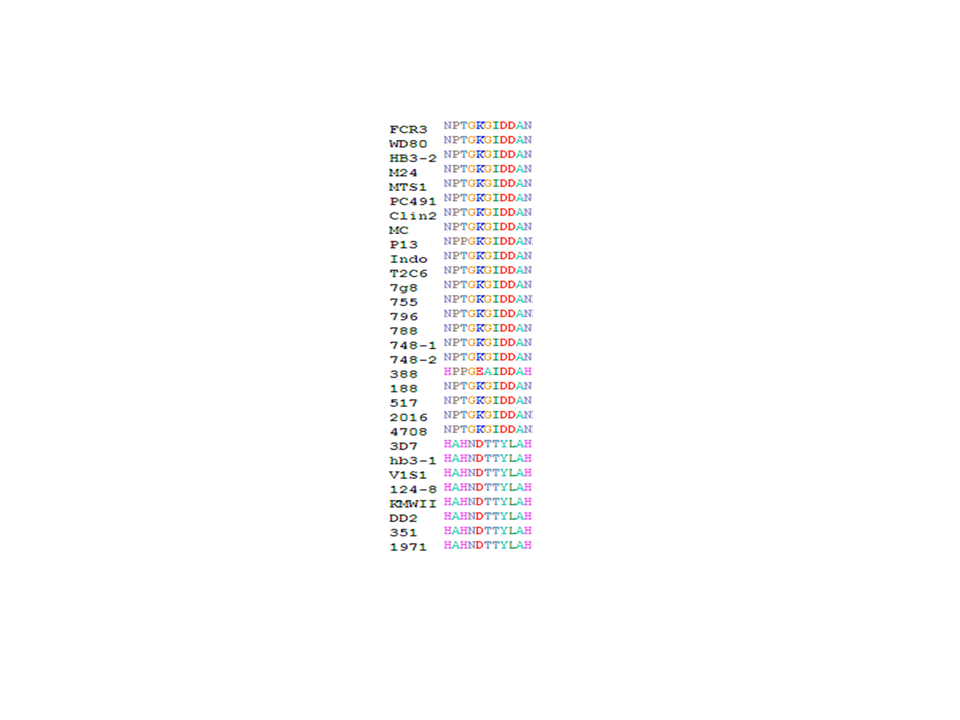

Supplement: Figure S2 — Comparison of 30 P. falciparum sequences covering the polymorphic region of peptide-region P22–P26 by alignment using BioEdit. (TIF) [file pone.0043663.s002.tif]
