# Supplementary material for: Identification and Characterization of B-Cell Epitopes in the DBL4ε Domain of VAR2CSA
Source: PLoS One. 2012 Sep 6;7(9):e43663. doi: 10.1371/journal.pone.0043663 (PMC3435390; doi:10.1371/journal.pone.0043663)
Supplement: Table S1 — Amino acid sequence of each peptide and corresponding amino acid position in the DBL4ε-FCR3 domain. (DOCX) [file pone.0043663.s003.docx]

**Table S1: Amino acid sequence of each peptide and corresponding amino acid position in the DBL4ε-FCR3 domain.**

| Peptide no. | Position | Amino Acid sequence | Peptide no. | Position | Amino Acid sequence |
| --- | --- | --- | --- | --- | --- |
| 1 | 25-40 | KKYIKKLENGRSLEGV | 32 | 118-133 | DIIKGNDLVHDEYTKYI |
| 2 | 28-43 | IKKLENGRSLEGVYVP | 33 | 127-142 | HDEYTKYIDSKLNEIF |
| 3 | 31-46 | LENGRSLEGVYVPPRR | 34 | 135-150 | DSKLNEIFGSSNTNDI |
| 4 | 34-49 | GRSLEGVYVPPRRQQL | 35 | 143-158 | GSSNTNDIDTKRARTD |
| 5 | 37-52 | LEGVYVPPRRQQLCLY | 36 | 151-166 | DTKRARTDWWENETIT |
| 6 | 40-55 | VYVPPRRQQLCLYELF | 37 | 159-174 | WWENETITNGTDRKTI |
| 7 | 43-58 | PPRRQQLCLYELFPII | 38 | 167-182 | NGTDRKTIRQLVWDAM |
| 8 | 46-61 | RQQLCLYELFPIIIKN | 39 | 175-190 | RQLVWDAMQSGVRYAV |
| 9 | 49-64 | LCLYELFPIIIKNEEG | 40 | 183-198 | QSGVRYAVEEKNENFP |
| 10 | 52-67 | YELFPIIIKNEEGMEK | 41 | 191-206 | EEKNENFPLCMGVEHI |
| 11 | 55-70 | FPIIIKNEEGMEKAKE | 42 | 199-214 | LCMGVEHIGIAKPQFI |
| 12 | 58-73 | IIKNEEGMEKAKEELL | 43 | 207-222 | GIAKPQFIRWLEEWTN |
| 13 | 61-76 | NEEGMEKAKEELLETL | 44 | 215-230 | RWLEEWTNEFCEKYTK |
| 14 | 64-79 | GMEKAKEELLETLQIV | 45 | 223-238 | EFCEKYTKYFEDMKSK |
| 15 | 67-82 | KAKEELLETLQIVAER | 46 | 231-246 | YFEDMKSKCDPPKRAD |
| 16 | 70-85 | EELLETLQIVAEREAY | 47 | 239-254 | CDPPKRADTCGDNSNI |
| 17 | 73-88 | LETLQIVAEREAYYLW | 48 | 247-262 | TCGDNSNIECKKACAN |
| 18 | 76-91 | LQIVAEREAYYLWKQY | 49 | 255-270 | ECKKACANYTNWLNPK |
| 19 | 79-94 | VAEREAYYLWKQYNPT | 50 | 263-278 | YTNWLNPKRIEWNGMS |
| 20 | 82-97 | REAYYLWKQYNPTGKG | 51 | 271-286 | RIEWNGMSNYYNKIYR |
| 21 | 85-100 | YYLWKQYNPTGKGIDD | 52 | 279-294 | NYYNKIYRKSNKESED |
| 22 | 88-103 | WKQYNPTGKGIDDANK | 53 | 286-302 | KSNKESEDGKDYSMIM |
| 23 | 91-106 | YNPTGKGIDDANKKAC | 54 | 295-310 | GKDYSMIMAPTVIDYL |
| 24 | 94-109 | TGKGIDDANKKACCAI | 55 | 303-318 | APTVIDYLNKRCHGEI |
| 25 | 97-112 | GIDDANKKACCAIRGS | 56 | 311-326 | NKRCHGEINGNYICCS |
| 26 | 100-115 | DANKKACCAIRGSFYD | 57 | 319-334 | NGNYICCSCKNIGAYN |
| 27 | 103-118 | KKACCAIRGSFYDLED | 58 | 327-342 | CKNIGAYNTTSGTVNK |
| 28 | 106-121 | CCAIRGSFYDLEDIIK | 59 | 335-350 | TTSGTVNKKLQKKETE |
| 29 | 109-124 | IRGSFYDLEDIIKGND | 60 | 343-358 | KLQKKETECEEEKGPL |
| 30 | 112-127 | SFYDLEDIIKGNDLVH | 61 | 351-366 | CEEEKGPLDLMNEVLN |
| 31 | 115-130 | DLEDIIKGNDLVHDEY | 62 | 359-374 | DLMNEVLNKMDKKYSA |
